# Outcomes of critically ill patients with newly diagnosed Burkitt’s lymphoma: a monocentric retrospective study

**DOI:** 10.1016/j.aicoj.2026.100084

**Published:** 2026-05-22

**Authors:** Fabiola Cammarota, Charlotte Degoutte, Laure Calvet, Dara Chean, Claire Fieschi, Catherine Thieblemont, Gennaro De Pascale, Michael Darmon, Lara Zafrani, Massimo Antonelli, Elie Azoulay, Thibault Dupont

**Affiliations:** a Medical Intensive Care Unit, Saint Louis Hospital, Assistance Publique Hôpitaux de Paris (APHP), Université de Paris Cité, Paris, France; b Dipartimento di Scienze Biotecnologiche di Base, Cliniche Intensivologiche e Perioperatorie, Università Cattolica del Sacro Cuore, Rome, Italy; c Dipartimento di Scienze dell'Emergenza, Anestesiologiche e della Rianimazione, Fondazione Policlinico Universitario A. Gemelli IRCCS, Rome, Italy; d Hematology Department, Le Mans Hospital, Le Mans, France; e Medical Intensive Care Unit, Clermont-Ferrand Hospital, Clermont-Ferrand, France; f Service de Réanimation Polyvalente, Centre Hospitalier de Laval, Laval, France; g Department of Clinical Immunopathology, Saint Louis Hospital, Assistance Publique Hôpitaux de Paris (APHP), Université de Paris Cité, Paris, France; h Department of Onco-Hematology, Saint Louis Hospital, Assistance Publique Hôpitaux de Paris (APHP), Université de Paris Cité, Paris, France

**Keywords:** Burkitt’s lymphoma, Tumor Lysis Syndrome, HIV

## Abstract

**Background:**

Burkitt’s lymphoma (BL) can lead to intensive care unit (ICU) admission at diagnosis and treatment initiation for management of disease-related complications such as tumor lysis syndrome (TLS) and acute kidney injury (AKI). Data on the outcomes and prognostic factors in this population of critically ill patients remain limited. We conducted a retrospective single-center study including 124 patients admitted to the ICU of Saint-Louis Hospital with newly diagnosed BL between January 1, 2002 and December 31, 2023. Univariate and multivariate survival analyses were performed to determine factors associated with 3 months and 12 months mortality.

**Results:**

124 patients with a median age of 47.5 years (IQR, 34–58.5) were included, of which 60% (74/124) had HIV infection and a majority had advanced stage disease. Chemotherapy was administered during the ICU stay in 84% (104/124) of patients and 59% (73/124) developed TLS. Ninety-day and one-year mortality were respectively 34% (42/124) and 50% (60/119). Among the 50% (60/119) of patients who survived at one year, 98% (59/60) had achieved complete remission and 90% (52/58) had good functional outcomes (performance status of 0 or 1). In multivariate analysis, TLS, vasopressors, HIV infection, male sex, and increasing age were independently associated with one-year mortality.

**Conclusions:**

Patients with BL admitted to the ICU during the early phase of their disease present with high rates of TLS, HIV co-infection, and organ failures. Despite low severity scores at admission, short-term and long-term survival remain poor, with half of patients dying within one year. However, among one-year survivors, nearly all achieved complete remission with good functional outcomes. TLS, vasopressor use, HIV infection, male sex, and increasing age were independently associated with one-year mortality.

## Background

Burkitt’s lymphoma (BL) is a type of aggressive B-cell non-Hodgkin lymphoma (NHL) that accounts for less than 5% of adult lymphomas [1]. Three distinct subtypes of BL have been described: the endemic form, commonly associated with Epstein-Barr virus (EBV) infection; the sporadic form, more prevalent in non-malaria-endemic regions; and the immunodeficiency-associated form, most frequently affecting patients living with human immunodeficiency virus (PLHIV) infection [2,3]. Among hematologic malignancies, BL exhibits the most rapid proliferation rate [4,5] and its aggressiveness is primarily driven by the t(8; 14) translocation leading to an overexpression of the *MYC* proto-oncogene. Clinically, BL may present with spontaneous tumor lysis syndrome (TLS), bone marrow or central nervous system (CNS) infiltration [6]. Bone marrow infiltration with more than 25% of blast cells defines Burkitt-type leukemia or L3 type acute lymphoblastic leukemia (L3-ALL) [6]. Given its marked chemosensitivity and the risk of acute complications related to TLS or compression syndromes, prompt treatment initiation is essential in BL management [7] with overall response rates to multi-agent chemotherapy reaching approximately 90% [[5], [6], [7], [8], [9], [10]].

Historically, patients with BL were considered to have poor outcomes, particularly PLHIV, although their prognosis in the ICU significantly improved over the years [[11], [12], [13], [14], [15], [16]]. Only a limited number of studies have evaluated ICU outcomes in patients with lymphoma, and only a few have specifically included patients with BL [14,15]. In a retrospective study, BL was independently associated with an increased risk of mortality [14], whereas another study reported that all BL patients survived their ICU stay, with overall response rates unaffected by an ICU stay.

To date, no study has specifically addressed the ICU course and outcomes of patients with Burkitt’s lymphoma. Therefore, we aimed to describe the clinical characteristics and outcomes of patients admitted to the ICU during initial management of BL, and factors associated with early ninety-day mortality and one-year outcomes.

## Materials and methods

### Study design and participants

**Fig. 1:**
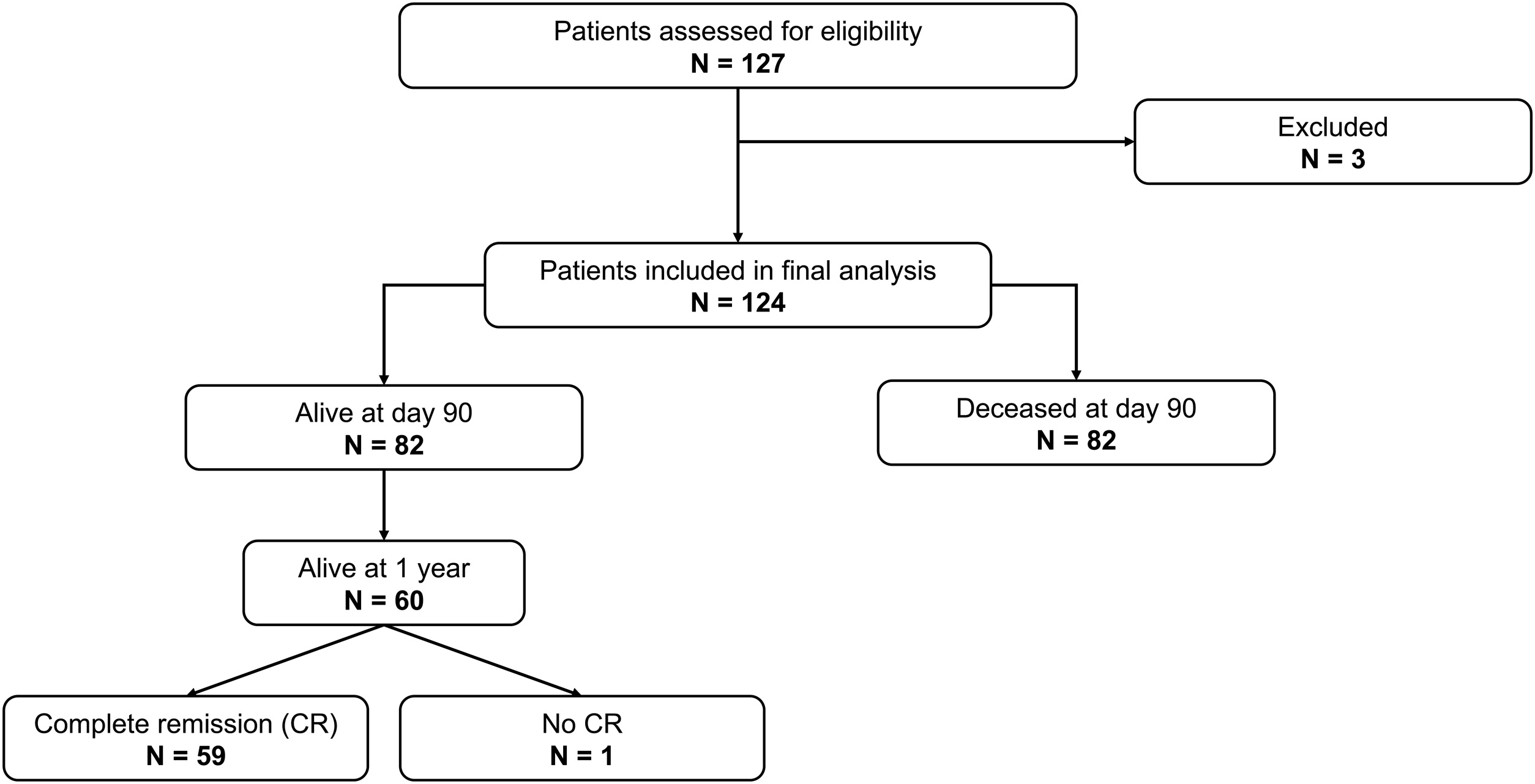
Flowchart of the study.

We conducted a retrospective, observational, single-center study, including all consecutive patients admitted to the medical ICU of Saint-Louis Hospital between January 1, 2002 and December 31, 2023 (Fig. 1, flow chart). Patients were included if they met the following criteria: a diagnosis of Burkitt lymphoma or type 3 acute lymphoblastic leukemia (ALL), and admission to the ICU for initial management of the hematologic malignancy, defined as an admission occurring before the second cycle of chemotherapy (excluding prephase cyclophosphamide, vincristine, prednisone – COP- therapy). Exclusion criteria included patients admitted to the ICU with a prior history of BL, and patients receiving first-line treatment for BL who were admitted to the ICU after the second cycle of chemotherapy (excluding COP). In cases of multiple ICU admissions during initial disease management, only the first ICU stay was considered. The primary outcome was 90-day mortality and the secondary outcome was 1-year mortality.

### Data collection & ethics

Data was collected by local investigators using electronic case report forms (eCRF), anonymized and centralized on a computer. We collected epidemiologic, demographic, medical history, and biologic data upon ICU admission. This study has been approved by the Ethics Committee of the French Intensive Care Society (FICS; IRB number: CE SRLF 26-023). Informed consent was waived due to the retrospective nature of the study. The study was conducted according to the Declaration of Helsinki principles.

### Definitions

The presence of significant comorbidities was defined based on the patient's medical history and included chronic obstructive pulmonary disease (COPD), chronic heart failure, chronic kidney disease, liver cirrhosis, diabetes mellitus, obesity, and a history of solid tumors, hematologic malignancies (other than Burkitt Lymphoma), or solid organ transplantation (SOT). The diagnosis of BL was established by histopathological examination of lymph node or extranodal tissue biopsies, in accordance with the World Health Organization (WHO) classification criteria [17]. Diagnostic confirmation required the presence of characteristic morphological features and a compatible immunohistochemical profile. Molecular diagnosis was performed by identifying a *MYC* gene rearrangement, in the absence of *BCL6* and *BCL2* rearrangements. When conventional cytogenetic analysis or fluorescence in situ hybridization (FISH) were not available, diagnosis relied solely on histomorphological and immunophenotypic findings. Disease staging was determined using the Ann Arbor classification [18,19]. Central nervous system (CNS) involvement was defined by the presence of lymphoma cells in a cerebrospinal fluid (CSF) analysis and/or clinical signs suggestive of CNS infiltration. Disease severity and organ dysfunction were assessed using the Simplified Acute Physiology Score II (SAPS II) [20] and the Sequential Organ Failure Assessment (SOFA) scores [21], respectively. Tumor lysis syndrome (TLS) was defined according to the Cairo-Bishop classification [22] and hemophagocytic lymphohistiocytosis (HLH) was defined based on the HLH-2004 diagnostic criteria [23]. Aplasia was defined as an absolute neutrophil count below 500/mm^3^. BL status at the most recent patient follow-up was categorized as complete remission (CR), partial remission (PR), relapse, or ongoing first-line treatment. CR was defined by clinical, biological, and radiological remission, along with the absence of excess blast cell in a bone marrow aspirate (if initially present) at various timepoints.

### Statistical analysis

Quantitative variables were described either as median with interquartile range (IQR, Q1-Q3) and compared using Wilcoxon’s rank sum test. Qualitative variables were described as count and percentages and compared using Fisher’s exact test. Survival functions were computed using Kaplan–Meier’s estimates on right-censored data and group comparison was performed using univariate log rank’s test for each subgroup. Overall survival (OS) was defined as time from ICU admission to death from any cause. Progression-free survival (PFS), was defined as time from ICU admission to relapse or death from any cause (Table S2, Figure S1, S3). For multivariate analysis, a Cox’s proportional hazards model was computed using a stepwise feature selection method based on the Akaike Information Criteria (AIC) starting from a full model including the following variables: HIV infection, sex, age, vasopressors, mechanical ventilation, RRT, TLS, and year of admission. The analysis was performed on complete cases only (n = 127, 61 events). No imputation of missing data was performed. An exploratory interaction analysis between TLS and RRT was tested in a separate model to assess whether the prognostic effect of TLS differed according to RRT status (Table S4). The proportional hazards assumption was assessed using Schoenfeld residuals (Table S5). Also, a sensitivity analysis was performed using a Cox model restricted to variables measured at baseline: HIV infection, age, SOFA score at admission, and year of admission (Table S6). Hazard ratios (HR) and their 95% confidence intervals (CI) are reported. A variance inflation factor (VIF) analysis to evaluate collinearity among candidate variables in the Cox model was also conducted (Table S7). All tests were two-sided, and a p-value of less than 0.05 was considered significant. All statistical analyses were carried out using R and the following packages: *dplyr*, *gtsummary*, *gt*, *survival, survminer, ggplot2.*

## Results

### Demographics and characteristics

During the study period, 127 patients were screened for BL and 124 were included (Fig. 1). Patients’ characteristics are summarized in Table 1 and S1. Median age was 47.5 years (IQR 34, 58.5) and most patients were male (80%, n = 99) with more than half having HIV infection (60%, n = 75). A total of 36% of patients (n = 46) had L3-ALL. The diagnosis of Burkitt lymphoma was based on bone marrow aspirate cytology in 36% (n = 46), lymph node biopsy in 20% (n = 26), and other biopsy sites in 43% (n = 55) of patients. Bone marrow and CNS involvement were respectively present in 69% (n = 85) and in 52% (n = 62) of patients. A vast majority of patients (94%, n = 116) had extensive stage IV disease according to the Ann Arbor classification. At ICU admission, the median Sequential Organ Failure Assessment (SOFA) score was 2 [1,5]. Tumor lysis syndrome (TLS) occurred in 59% of patients (n = 73). Chemotherapy was administered in the ICU in 84% (n = 107) of patients (Table S1).

**Table 1:** Patient’s characteristics and univariate analysis according to 90-day mortality.

Variable	N	Alive N = 82	Deceased N = 42	p-value^1^
Age, Median [Q1, Q3]	124	41.00 [30.00, 53.00]	54.00 [42.00, 63.00]	<0.001
Male Sex, n / N (%)	124	63 / 82 (77%)	36 / 42 (86%)	0.24
Comorbidities, n / N (%)	124			0.84
Chronic Kidney Disease		1 / 82 (1.2%)	1 / 42 (2.4%)	
Cirrhosis		1 / 82 (1.2%)	0 / 42 (0%)	
COPD		2 / 82 (2.4%)	0 / 42 (0%)	
No		60 / 82 (73%)	30 / 42 (71%)	
Other		18 / 82 (22%)	11 / 42 (26%)	
HIV infection, n / N (%)	124	44 / 82 (54%)	30 / 42 (71%)	0.056
Diagnosis, n / N (%)	124			0.16
Burkitt		48 / 82 (59%)	30 / 42 (71%)	
L3-ALL		34 / 82 (41%)	12 / 42 (29%)	
Type of biopsy, n / N (%)	124			0.26
Lymph Node		19 / 82 (23%)	6 / 42 (14%)	
Medullar		32 / 82 (39%)	14 / 42 (33%)	
Other		31 / 82 (38%)	22 / 42 (52%)	
Caryotype, n / N (%)	77			0.013
No medullar involvement		10 / 56 (18%)	2 / 21 (9.5%)	
No translocation		4 / 56 (7.1%)	8 / 21 (38%)	
t (8;14)		39 / 56 (70%)	10 / 21 (48%)	
t (8;22)		3 / 56 (5.4%)	1 / 21 (4.8%)	
cMyc rearrangement - FISH, n / N (%)	63			0.054
No		4 / 44 (9.1%)	6 / 19 (32%)	
Yes		40 / 44 (91%)	13 / 19 (68%)	
Ann Arbor's classification, n / N (%)	124			0.27
1		1 / 82 (1.2%)	0 / 42 (0%)	
2		2 / 82 (2.4%)	0 / 42 (0%)	
3		5 / 82 (6.1%)	0 / 42 (0%)	
4		74 / 82 (90%)	42 / 42 (100%)	
Medullar infiltration, n / N (%)	123	54 / 81 (67%)	30 / 42 (71%)	0.59
CNS involvement, n / N (%)	118	40 / 81 (49%)	21 / 37 (57%)	0.46
Reason of Admission, n / N (%)	119			0.014
Acute Kidney Injury		10 / 79 (13%)	4 / 40 (10%)	
Acute Respiratory Failure		5 / 79 (6.3%)	8 / 40 (20%)	
Coma		0 / 79 (0%)	1 / 40 (2.5%)	
Monitoring		41 / 79 (52%)	9 / 40 (23%)	
Other cause		7 / 79 (8.9%)	4 / 40 (10%)	
Shock		4 / 79 (5.1%)	3 / 40 (7.5%)	
Tumor Lysis Syndrome		12 / 79 (15%)	11 / 40 (28%)	
SOFA Score - Admission, Median [Q1, Q3]	124	2.00 [0.00, 4.00]	3.00 [1.00, 7.00]	0.009
Chemotherapy in ICU, n / N (%)	124	71 / 82 (87%)	33 / 42 (79%)	0.25
Tumor lysis syndrome, n / N (%)	124	41 / 82 (50%)	32 / 42 (76%)	0.005
Hemophagocytic Syndrome, n / N (%)	124	2 / 82 (2.4%)	2 / 42 (4.8%)	0.60
Aplasia, n / N (%)	124	19 / 82 (23%)	22 / 42 (52%)	0.001
Vasopressors, n / N (%)	124	5 / 82 (6.1%)	23 / 42 (55%)	<0.001
Mechanical ventilation, n / N (%)	124	8 / 82 (9.8%)	24 / 42 (57%)	<0.001
Non-invasive ventilation, n / N (%)	124	3 / 82 (3.7%)	3 / 42 (7.1%)	0.41
RRT, n / N (%)	124	32 / 82 (39%)	33 / 42 (79%)	<0.001
Length of ICU stay (days), Median [Q1, Q3]	124	4.00 [2.00, 7.00]	7.50 [3.00, 14.00]	0.021
Delay diagnosis-admission (days), Median [Q1, Q3]	124	3.50 [0.00, 14.00]	4.00 [1.00, 12.00]	0.70
Year of Admission, n / N (%)	124			0.014
2014 and before		46 / 82 (56%)	33 / 42 (79%)	
2015 and after		36 / 82 (44%)	9 / 42 (21%)	

1 Wilcoxon rank sum test; Pearson's Chi-squared test; Fisher's exact test.

### ICU management and 90 day mortality

Among the 124 patients in our cohort, the most frequent reason for ICU admission was monitoring (42%, n = 50), followed by acute kidney injury (12%, n = 14) and acute respiratory failure (11%, n = 13) (Table S1). Also, vasopressor support was required in 23% of patients (n = 28), mechanical ventilation (MV) in 26% (n = 32), and renal replacement therapy (RRT) in 52% (n = 65). Organ support was more frequent in non-survivors with vasopressor support in 55% (n = 23) of non-survivors versus 6.1% (n = 5) of survivors, MV in 57% (n = 24) versus 9.8% (n = 8), and RRT in 79% (n = 33) versus 39% (n = 32). At 90 days, 82 (66%) were alive while 42 (34%) had died (Table 1).

### Long-term survival and functional outcomes at one year

Of the 124 patients, 119 had available one-year follow-up data. At one-year follow-up, 50% of patients (n = 60/119) were alive (Figure S1). Among the 58 patients for whom performance status was available at one year, 52% (n = 30/58) had a score of 0, 38% (n = 22/58) a score of 1, and 10% (n = 6/58) a score of 2, yielding an overall good functional outcome rate (PS 0 or 1) of 90% (52/58). Of the 60 patients assessed for disease status at one year, 98% (n = 59/60) had achieved complete remission (CR).

Long-term survival outcomes varied markedly across clinical subgroups (Table 2 & S2). At one-year, HIV-negative patients showed significantly higher overall survival (OS) (Table 2) compared with HIV-positive patients (OS: 68% vs. 38%, *p* = 0.003). Neither sex nor diagnosis (Burkitt’s lymphoma vs. L3-ALL) was associated with differences in OS. Requirement for vasopressors, MV, or RRT was associated with markedly reduced survival. At one year, OS was 18% (95% CI, 8.1–40%) with vasopressors vs. 60% (95% CI, 51–71%) without vasopressors, 18% (95% CI, 8.3–38%) vs. 61% (95% CI, 52–72%) for MV, and 35% (95% CI, 25–49%) vs. 67% (95% CI, 56–80%) for RRT (all *p* < 0.001).

**Table 2:** Overall Survival (OS) probability at 3, 6 and 12 months by subgroup.

Characteristic^1^	3 Months^1^	6 Months^1^	12 Months^1^	p-value^1^
HIV infection				0.003
HIV −	76% (65%, 89%)	72% (61%, 86%)	68% (56%, 82%)	
HIV +	60% (49%, 72%)	51% (41%, 64%)	38% (29%, 51%)	
Sex				0.91
Male	64% (55%, 74%)	61% (52%, 71%)	50% (41%, 61%)	
Female	76% (61%, 95%)	55% (39%, 79%)	51% (35%, 75%)	
Diagnosis				0.78
Burkitt's lymphoma	62% (52%, 74%)	59% (49%, 71%)	50% (40%, 62%)	
L3-ALL	74% (62%, 88%)	61% (48%, 77%)	52% (39%, 69%)	
Vasopressors				<0.001
Vasopressors −	80% (73%, 89%)	72% (63%, 81%)	60% (51%, 71%)	
Vasopressors +	18% (8.1%, 40%)	18% (8.1%, 40%)	18% (8.1%, 40%)	
Mechanical Ventilation				<0.001
MV −	81% (73%, 89%)	72% (63%, 82%)	61% (52%, 72%)	
MV +	25% (14%, 46%)	25% (14%, 46%)	18% (8.3%, 38%)	
RRT				<0.001
RRT −	85% (76%, 94%)	78% (68%, 89%)	67% (56%, 80%)	
RRT +	49% (39%, 63%)	43% (33%, 57%)	35% (25%, 49%)	
Tumor Lysis Syndrome				<0.001
				
TLS −	80% (70%, 92%)	78% (68%, 91%)	72% (61%, 86%)	
TLS +	56% (46%, 69%)	47% (36%, 60%)	35% (26%, 48%)	
Tumor Lysis Syndrome & RRT				0.002
TLS −	80% (70%, 92%)	78% (68%, 91%)	72% (61%, 86%)	
TLS+/RRT−	77% (57%, 100%)	52% (30%, 89%)	26% (9.9%, 68%)	
TLS+/RRT+	52% (41%, 66%)	45% (34%, 60%)	37% (26%, 51%)	
Year of Admission				0.11
Before 2014 (included)	59% (49%–70%)	53% (44%–66%)	46% (36%–58%)	
After 2014	80% (69%–93%)	71% (59%–86%)	59% (45%–75%)	

1 Survival rates reported as % (95% CI).

**Fig. 2:**
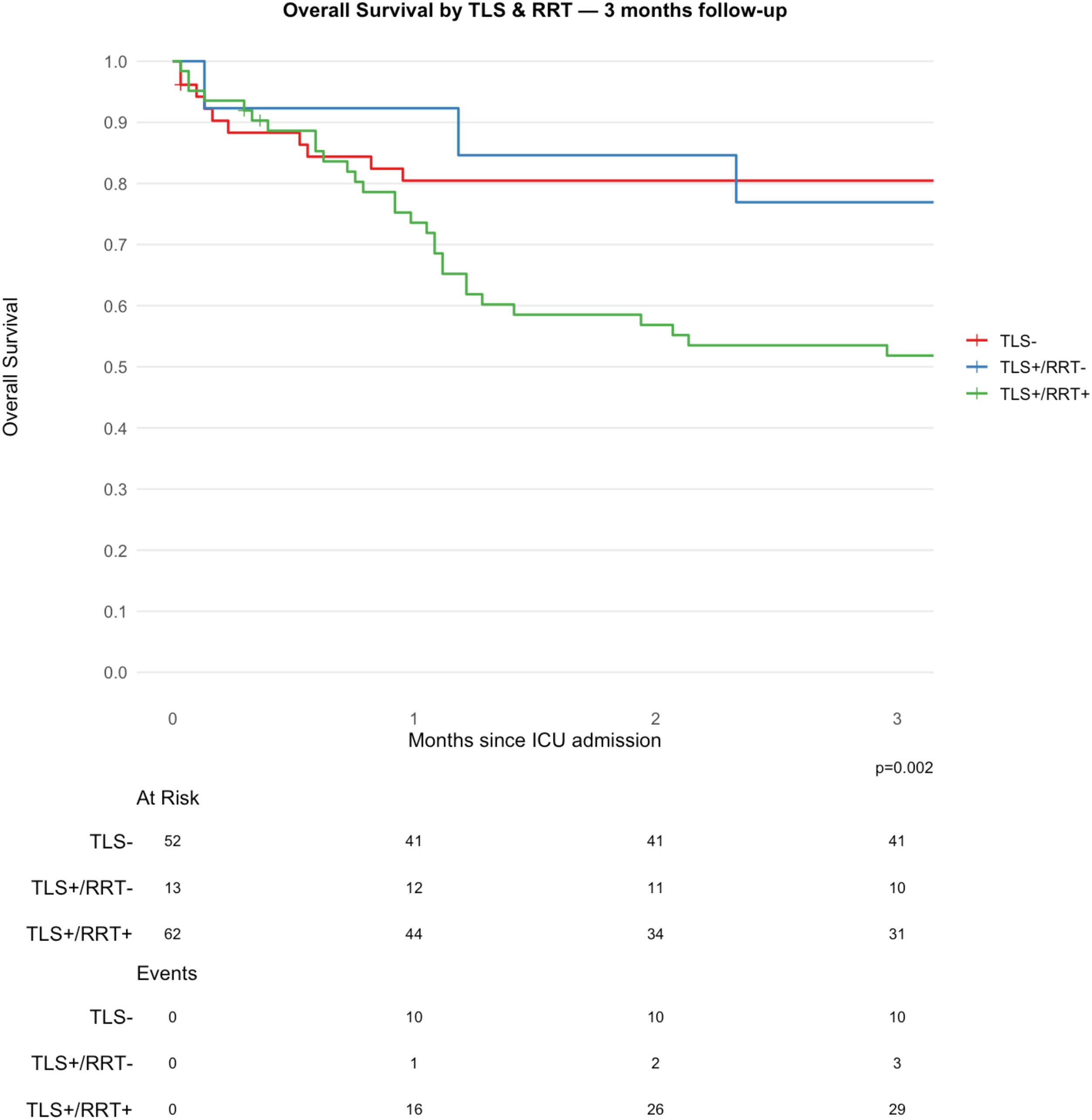
Overall Survival according to TLS and RRT at 3 months follow-up. TLS: Tumor Lysis Syndrome; RRT: Renal Replacement Therapy.

TLS was associated with poorer outcomes (OS: 35% [95% CI, 26–48%] vs. 72% [95% CI, 61–86%], *p* < 0.001). Patients with both TLS and RRT exhibited the lowest survival rates (OS: 37% [95% CI, 26–51%]) (Fig. 2, Table 2) as compared with those without TLS or RRT (OS: 72% [95% CI, 61–86%], *p* = 0.002). PFS estimates are reported in Table S2.

### Factors associated with survival at one-year

**Fig. 3:**
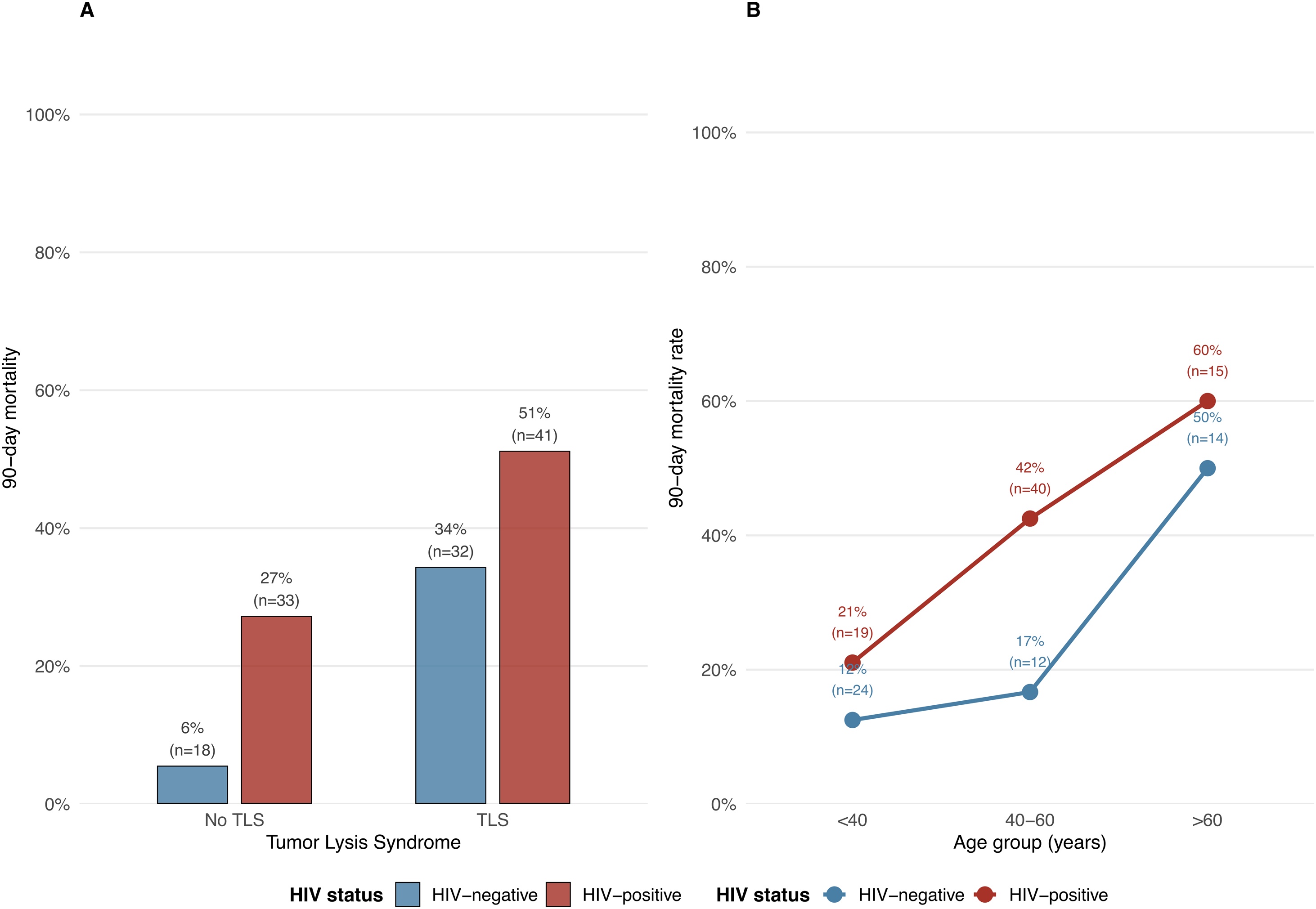
Ninety day mortality by TLS status (A) and age group (B) stratified by HIV status.

In multivariate analysis performed on complete-cases (n = 127, 61 events), factors independently associated with one-year mortality included: TLS (HR 3.83, 95% CI 1.95–7.54; p < 0.001) (Fig. 3A), vasopressor use (HR 4.11, 95% CI 1.87–9.03; p < 0.001), HIV infection (HR 2.51, 95% CI 1.38–4.57; p = 0.003), male sex (HR 2.04, 95% CI 1.00–4.15; p = 0.049), and increasing age (HR 1.13 per 5 years, 95% CI 1.02–1.25; p = 0.015) (Table 3, Fig. 3B). MV was not associated with one year mortality (HR 1.99, 95% CI 0.96–4.12; p = 0.066). The proportional hazards assumption was not met for vasopressor use and TLS (p = 0.007 and p = 0.0005 respectively). A potential interaction was observed between TLS and RRT (HR = 0.26; 95% CI 0.06–1.11) (Table S4). Also, in a sensitivity analysis restricted to baseline variables only HIV infection (HR 2.09, 95%CI [1.18–3.71], p = 0.012), age (HR 1.16 per 5 years, 95%CI [1.05–1.28], p = 0.002), and SOFA score at admission (HR 1.12 per point, 95%CI [1.06–1.18], p < 0.001) were independently associated with one-year mortality (Table S6).

**Table 3:** Risk factors for one-year mortality using a multivariate Cox model.

Characteristic	HR (95% CI)	p-value
HIV infection^1^	2.51 (1.38–4.57)	**0.003**
Male Sex^1^	2.04 (1.00–4.15)	**0.049**
Age (per 5 years)^1^	1.13 (1.02–1.25)	**0.015**
Vasopressors^1^	4.11 (1.87–9.03)	**<0.001**
Mechanical Ventilation^1^	1.99 (0.96–4.12)	0.066
Tumor Lysis Syndrome^1^	3.83 (1.95–7.54)	**<0.001**
Year of Admission^1^		
2014 and before^1^	—	
2015 and after^1^	0.58 (0.32–1.05)	0.070

Abbreviations: CI = Confidence Interval, HR = Hazard Ratio.

1 Complete case analysis: n = 127, events = 61, EPV = 8.7.

## Discussion

In this cohort of critically ill patients with Burkitt’s lymphoma, early 90-day and one-year mortality were 34% and 50%, respectively. TLS, vasopressor use, HIV infection, male sex, and increasing age were independently associated with higher one-year mortality. Among the 50% of patients who survived at one year, nearly all achieved CR with good functional outcomes (ECOG performance status 0 or 1).

To date, prior studies on BL outcomes in non-ICU hematology patients have reported overall survival rates ranging from 70% at 2 years [24] to 74–83% at 3 years [25]. By contrast, survival in HIV-positive patients was lower, with 2-year OS around 47% [26]. When compared with non-ICU hematology studies in non-HIV patients with Burkitt’s lymphoma, our survival rates appear less favorable. In general lymphoma patients, Algrin et al. [14] reported ICU, hospital, and one-year mortality rates of 22%, 37%, and 51%, respectively, which are notably similar to our results. Importantly, Burkitt’s lymphoma was identified as an independent predictor of hospital mortality. Similarly, Schellongowski et al. [15], showed that among 23% of Burkitt’s lymphoma patients required ICU admission, mostly for early monitoring. All patients survived the ICU stay, and long-term survival did not differ significantly between ICU and non-ICU patients. By contrast, Ferrè et al. [27] observed a markedly higher one-year mortality exceeding 80% in 48 ICU patients with lymphoma, including both HIV-positive and HIV-negative cases with Burkitt’s lymphoma, however their cohort included patients with relapsed disease or prior stem-cell transplantation. More recently, a retrospective study of patients with acute lymphoblastic leukemia or Burkitt’s lymphoma reported that all Burkitt patients admitted to the ICU survived, and their long-term outcomes were not inferior to those managed without ICU admission [28]. In another analysis focusing on older adults with Burkitt lymphoma, ICU admission occurred in over 20% of cases, and mortality during treatment was disproportionately higher in patients aged ≥70 years, highlighting the impact of age on the outcome [29]. These findings are in line with our results, showing that increasing age emerged as an independent predictor of one-year mortality. Finally, recent population-based studies in hematologic malignancies have confirmed the high risk of ICU admission and significant mortality among patients with aggressive lymphomas [30]. Importantly, interruption or inability to resume chemotherapy after ICU admission has been shown to negatively affect long-term outcomes [31]. This emphasizes the need for early multidisciplinary management to ensure continuity of chemotherapy, even in the ICU setting. In our study, patients presented with limited organ dysfunction at admission, as reflected by a median SOFA score of 2. This finding explains the relatively low use of organ support compared with previously published studies and reflects local practices of early ICU admission for this high-risk patient population. Indeed, the most frequent reason for ICU admission was monitoring during initiation of the first cycle of chemotherapy and TLS prevention. While admitting patients without overt organ failure may appear excessive, close clinical and biological monitoring can be better ensured in the ICU setting. Such monitoring is particularly necessary in Burkitt’s lymphoma because of its highly aggressive nature, with a high risk of tumor lysis syndrome (TLS) and acute renal failure at treatment initiation.

This is consistent with our finding that dialysis was required more frequently in our cohort compared with other ICU lymphoma series [15,32]. Early management of organ dysfunction, particularly the renal one, is crucial as it directly impacts the feasibility of continuing chemotherapy at full dose intensity, thereby influencing long-term prognosis. Several studies have suggested the benefit of early ICU admission for patients with hematologic malignancies [16,28,29]. In our study, 84% of patients received initial chemotherapy in the ICU. Previous studies of patients with hematologic malignancies receiving chemotherapy in the ICU have reported that one third had concomitant infection at chemotherapy initiation [12,31] 54–62% required intubation, and 24–39% required dialysis [12,30,32]. Importantly, in the prospective study by Darmon et al. [30], concomitant infection at chemotherapy initiation was not independently associated with poor outcome. Collectively, these findings suggest the feasibility of initiating chemotherapy in the ICU, even in the presence of organ failure or active infection. In our analysis, both age and the presence of organ dysfunction were associated with increased mortality, a finding also reported by Algrin et al. [14]. This result is consistent with prior hematology and ICU studies, which have shown that the number and progression of organ failures are major prognostic factors [30,32,33].

HIV infection was independently associated with higher mortality in our cohort. In a retrospective study by Ferrè et al. [27] HIV infection had no impact on short-term mortality, whereas a study by Galicier et al. [26] reported lower overall survival in HIV-positive BL patients.

Furthermore, treatment-related toxicity may be higher in immunocompromised patients, contributing to mortality; in our cohort, nearly 75% of deaths among HIV-positive patients were treatment-related. Although RRT was associated with mortality in univariate analysis, it was not kept in the final multivariate model, likely reflecting collinearity with TLS. This is in line with previous studies reporting higher mortality among patients with acute renal failure compared with those without [29]. Similarly, Canet et al. [34] demonstrated that acute renal failure in patients with newly diagnosed hematologic malignancies was associated with a lower CR rate. Also, the interaction analysis suggested that the association between RRT and mortality may differ according to TLS status (Table S4).

Our study has several limitations. First, owing to its retrospective monocentric design, our findings may not be generalizable to other settings, particularly given the high proportion of PLHIV in our cohort and our early ICU admission policy. Second, this study was conducted over a large period spanning from 2002 to 2023, during which ICU practice, supportive care, chemotherapy regimens for BL and HIV management have evolved. Although we adjusted for admission period in our multivariate model, other residual confounding related to practice changes over this period cannot be excluded. Third, important biological parameters such as LDH and lactate levels that reflect tumor burden or the existence of a Warburg effect [35], which is associated with 1-year mortality were not collected in our database. Similarly, detailed chemotherapy regimens or dose intensity are not reported in our cohort. This may limit the interpretation of disease severity. Fourth, the proportional hazards assumption was not met for vasopressor use and TLS (Table S5) suggesting their prognostic impact was strongest in the early follow-up period. Therefore, the reported HR should be interpreted as average effects over the one-year follow-up period. Also, we lack a sensitivity analysis with a matched control population with non-Burkitt’s lymphoma that could strengthen our findings.

## Conclusion

In conclusion, patients with BL admitted to the ICU for inaugural disease management have a 50% one-year overall survival rate. Among patients alive at one year, 98% had achieved complete remission and 90% had good functional outcomes (performance status 0 or 1). Main independent predictors of one-year mortality were TLS, vasopressor use, HIV infection, male sex, and increasing age. Furthermore, HIV-positive patients face a substantial survival disadvantage. These results highlight the need for tailored critical care strategies for the high-risk HIV-positive subgroup.

## Authors' contributions

FC, CD, LC, EA, TD analyzed and interpreted the patient data. FC, EA, CD, TD performed statistical analysis. FC, CD, EA, TD wrote the initial draft. All authors read and approved the final manuscript.

## Consent for publication

Not applicable.

## Ethics approval and consent to participate

This study has been approved by the Ethics Committee of the French Intensive Care Society (FICS; IRB number: CE SRLF 26-023). Informed consent was waived due to the retrospective nature of the study. The study was conducted according to the Declaration of Helsinki principles.

## Funding

None.

## Availability of data and material

The datasets used and/or analysed during the current study are available from the corresponding author on reasonable request.

## Declaration of competing interest

The authors declare that they have no competing interests
