# Supplementary material for: Outcomes of critically ill patients with newly diagnosed Burkitt’s lymphoma: a monocentric retrospective study
Source: Ann Intensive Care. 2026 May 22;16:100084. doi: 10.1016/j.aicoj.2026.100084 (PMC13218120; doi:10.1016/j.aicoj.2026.100084)
Supplement: Supplementary file 1 [file mmc1.docx]

**Urgent Chemotherapy in Critically ill Patients with Highly Aggressive Burkitt’s Lymphoma**

Fabiola Cammarota, Charlotte Degoutte, Laure Calvet, Dara Chean, Claire Fieschi, Catherine Thieblemont, Gennaro De Pascale, Michael Darmon, Lara Zafrani, Massimo Antonelli, Elie Azoulay, Thibault Dupont

**Supplementary Material**

Table of Contents

[Figure S1: (A) Progression free survival (PFS) and Overall Survival (OS) at 24 months follow up 3](#_Toc227454030)

[Figure S2: Spline regression with (A) Year of admission (B) Duration of ICU stay and (C) age at admission vs. predicted mortality at 90 days 5](#_Toc227454031)

[Figure S3: Overall Survival (OS) and Progression Free Survival (PFS) at 12 months according to TLS, RRT status 8](#_Toc227454032)

[Figure S4: Overall Survival (OS) and Progression Free Survival (PFS) at 12 months according year of admission 10](#_Toc227454033)

[Figure S5: Progression Free Survival (PFS) probability according to TLS and RRT status at 12 months follow-up 12](#_Toc227454034)

[Table S1: Patient’s overall characteristics 13](#_Toc227454035)

[Table S2: Progression free survival probability at 3, 6 and 12 months by subgroup 15](#_Toc227454036)

[Table S3: Patients’ characteristics according to HIV status 16](#_Toc227454037)

[Table S4: Risk factors for one year mortality using a multivariate Cox model with an interaction term between RRT and TLS 18](#_Toc227454038)

[Table S5: Proportional hazards assumption — Schoenfeld residuals test 19](#_Toc227454039)

[Table S6: Sensitivity analysis — Cox model restricted to baseline predictors only 20](#_Toc227454040)

[Table S7: Variance Inflation Factors (VIF) analysis 21](#_Toc227454041)

## **Figure S1:** (A) Progression free survival (PFS) and Overall Survival (OS) at 24 months follow up

**A**

**
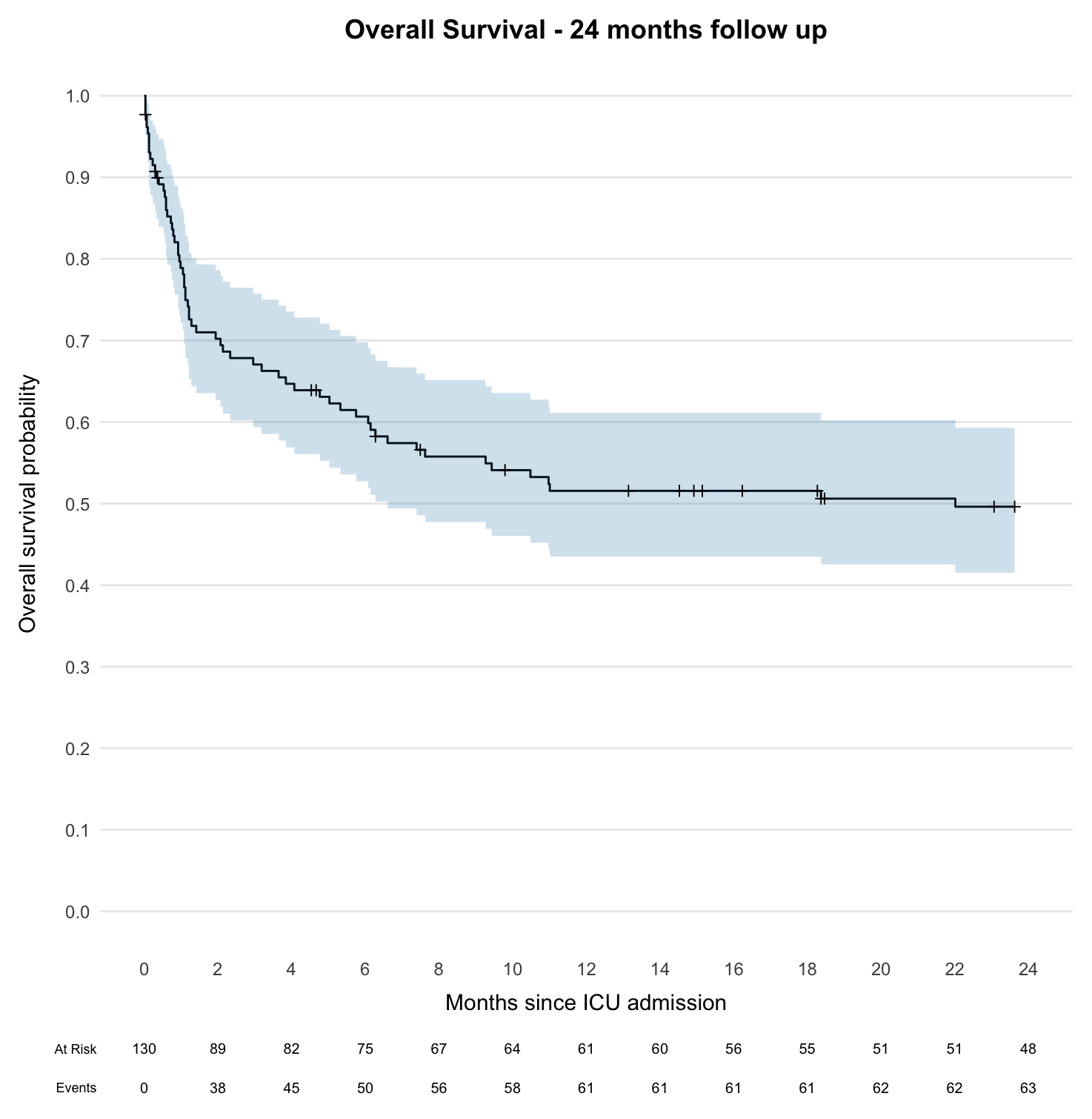
**

**B**

**
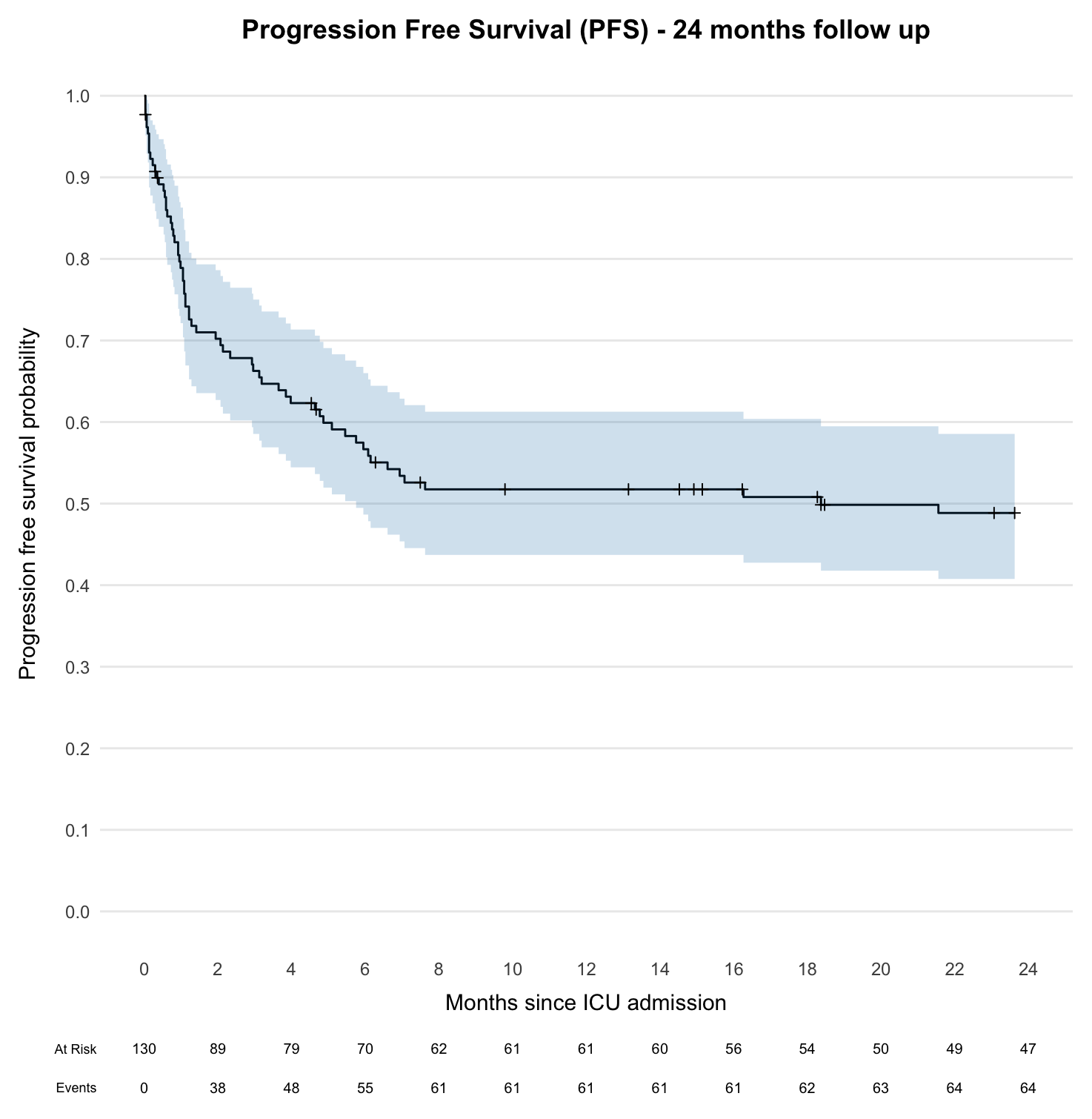
**

## **Figure S2:** Spline regression with (**A**) Year of admission (**B)** Duration of ICU stay and (**C)** age at admission vs. predicted mortality at 90 days

**A**








**
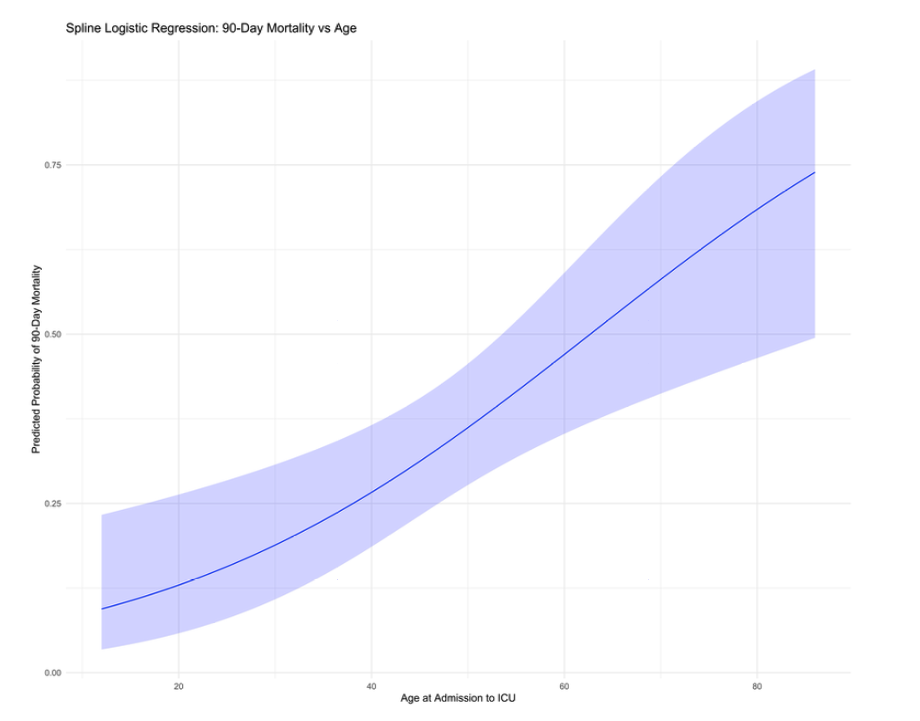
**

## **Figure S3**: Overall Survival (OS) and Progression Free Survival (PFS) at 12 months according to TLS, RRT status

**
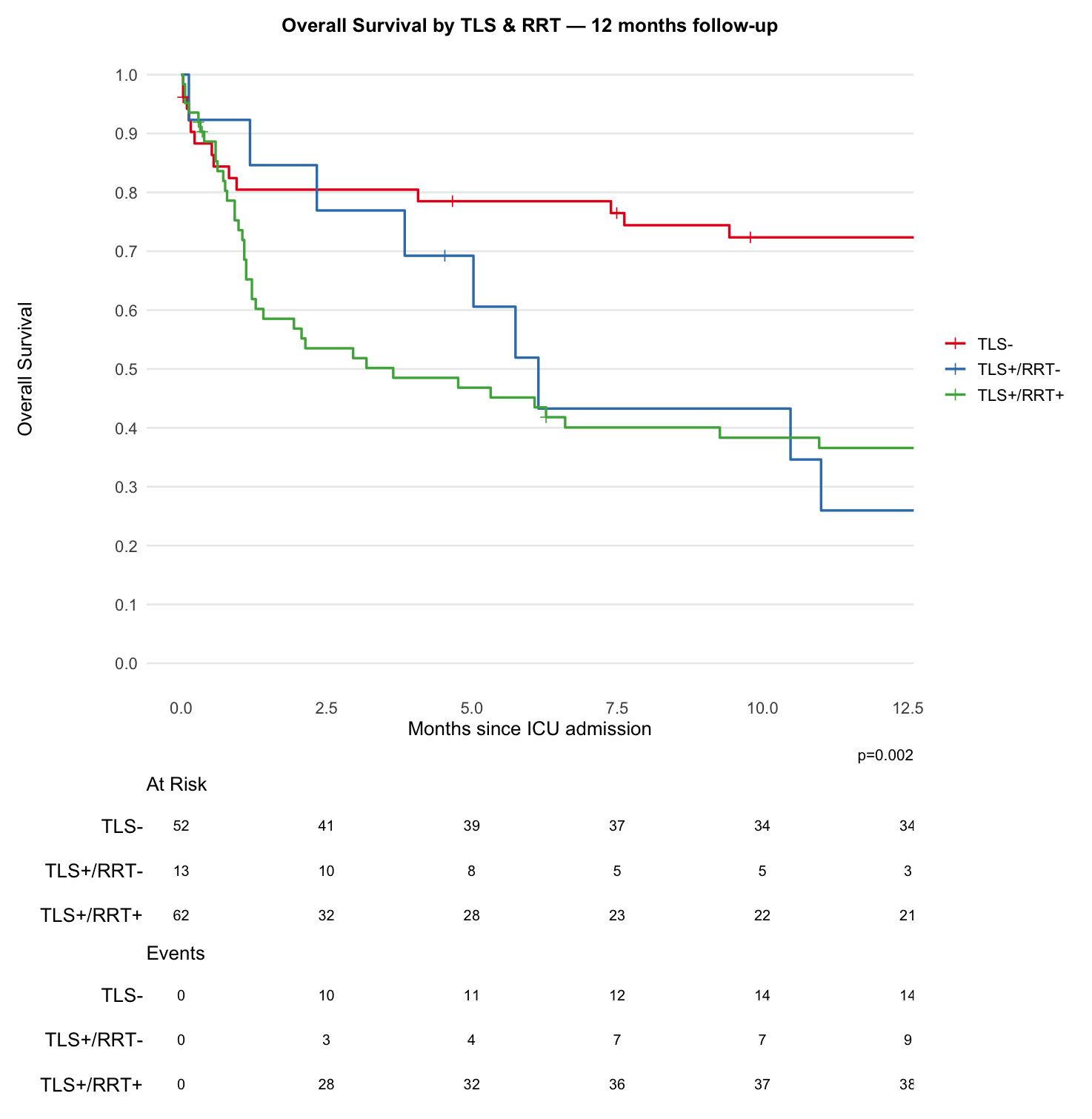
**

**
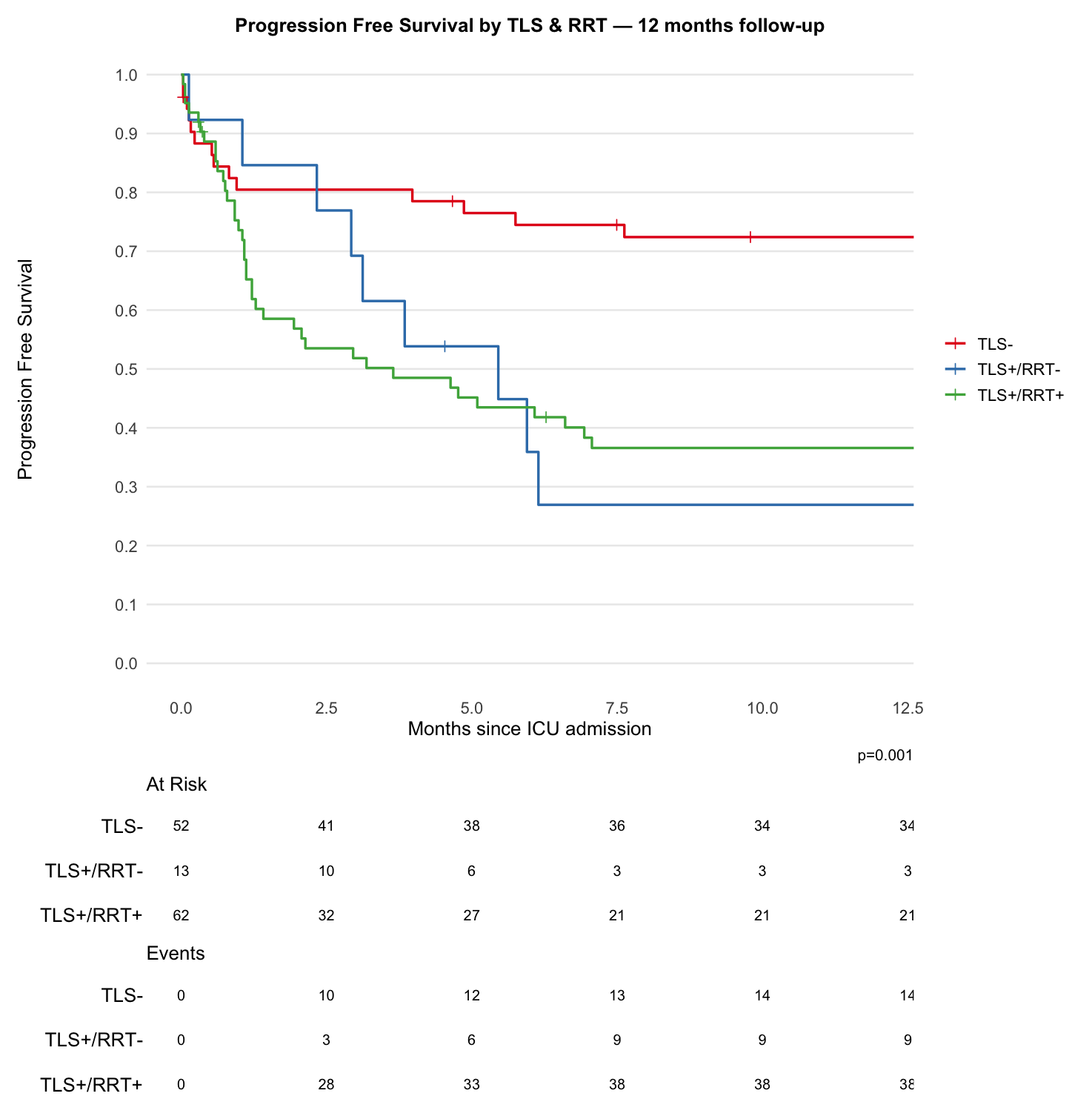
**

## **Figure S4**: Overall Survival (OS) and Progression Free Survival (PFS) at 12 months according year of admission

**
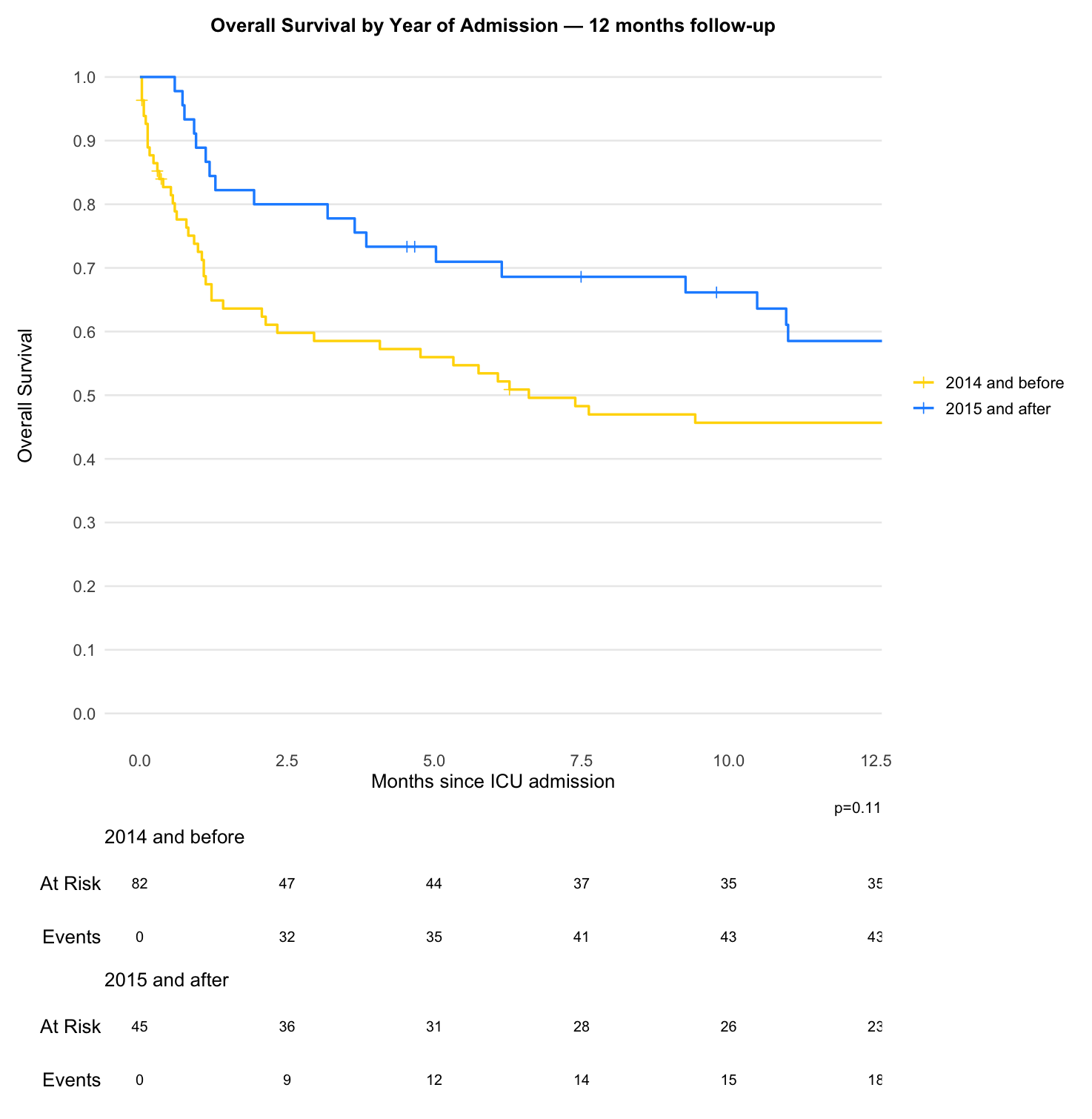
**

**
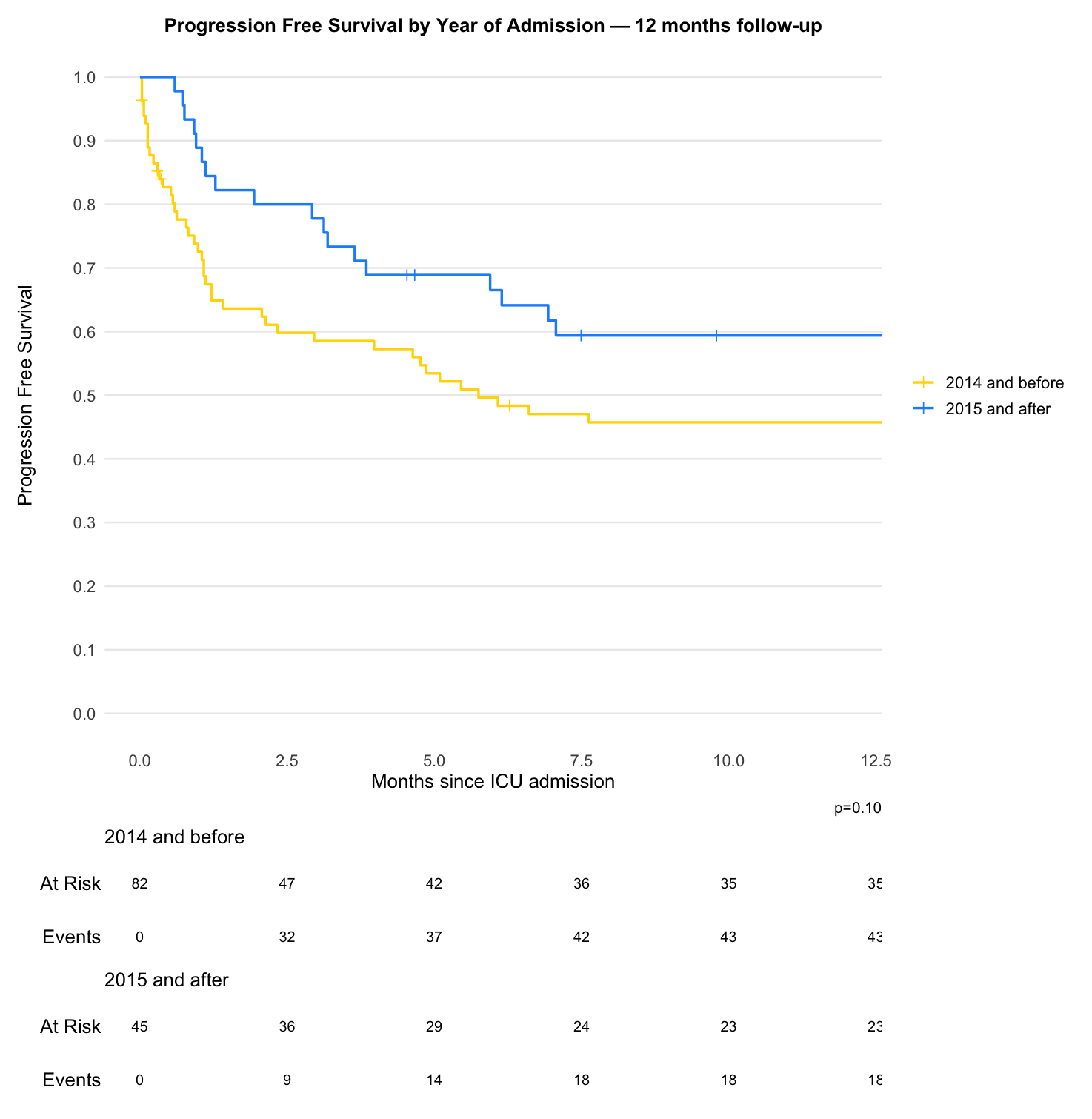
**

## **Figure S5**: Progression Free Survival (PFS) probability according to TLS and RRT status at 12 months follow-up


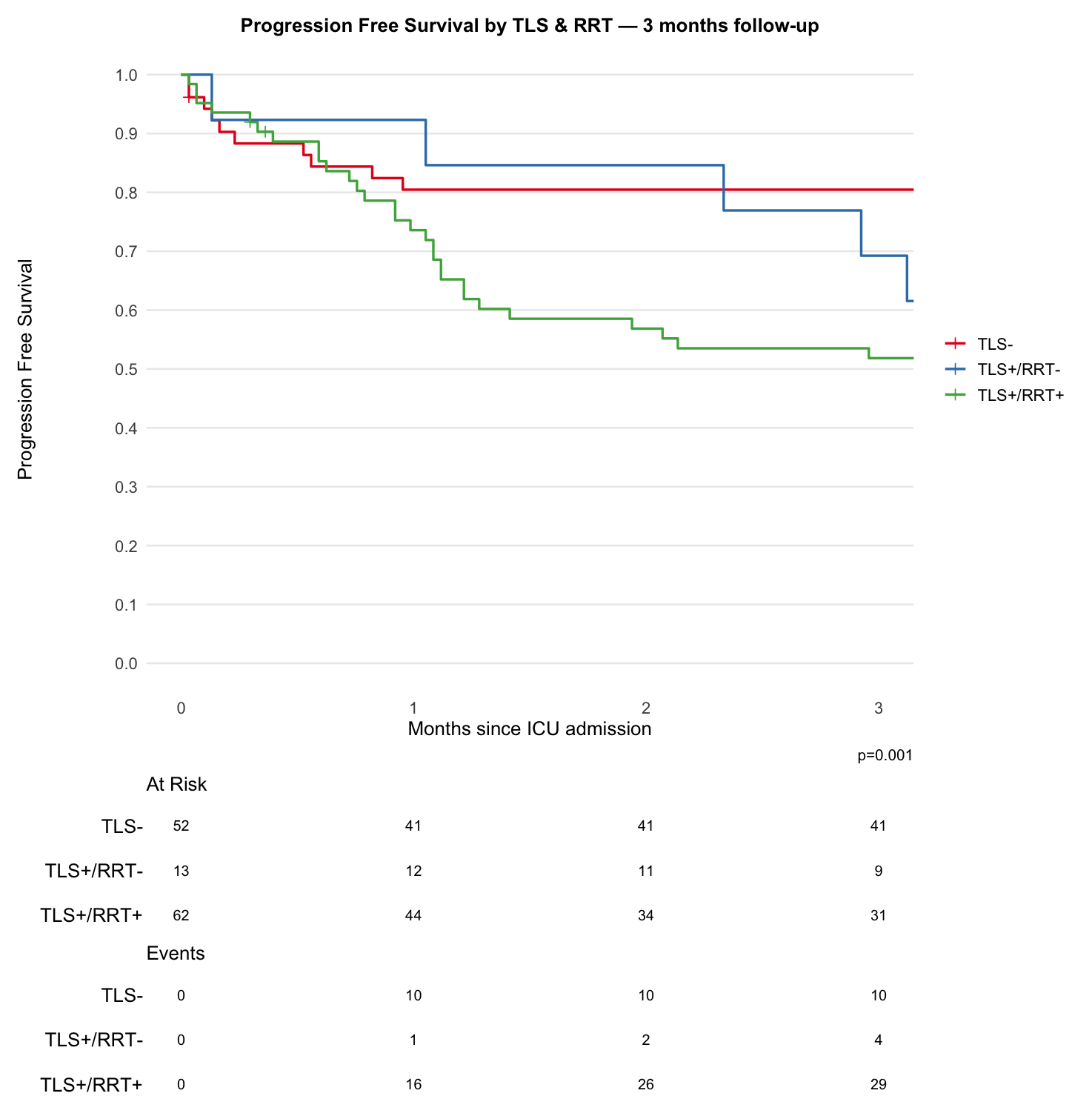


## **Table S1:** Patient’s overall characteristics

| **Variable** | **N** | **N = 124** |
| --- | --- | --- |
| **Age, Median [Q1, Q3]** | 124 | 47.50 [34.00, 58.50] |
| **Male Sex, n / N (%)** | 124 | 99 / 124 (80%) |
| **Comorbidities, n / N (%)** | 124 |  |
| Chronic Kidney Disease |  | 2 / 124 (1.6%) |
| Cirrhosis |  | 1 / 124 (0.8%) |
| COPD |  | 2 / 124 (1.6%) |
| No |  | 90 / 124 (73%) |
| Other |  | 29 / 124 (23%) |
| **HIV infection, n / N (%)** | 124 | 74 / 124 (60%) |
| **Diagnosis, n / N (%)** | 124 |  |
| Burkitt |  | 78 / 124 (63%) |
| L3-ALL |  | 46 / 124 (37%) |
| **Type of biopsy, n / N (%)** | 124 |  |
| Lymph Node |  | 25 / 124 (20%) |
| Medullar |  | 46 / 124 (37%) |
| Other |  | 53 / 124 (43%) |
| **Caryotype, n / N (%)** | 77 |  |
| No medullar involvement |  | 12 / 77 (16%) |
| No translocation |  | 12 / 77 (16%) |
| t(8;14) |  | 49 / 77 (64%) |
| t(8;22) |  | 4 / 77 (5.2%) |
| **cMyc rearrangement - FISH, n / N (%)** | 63 |  |
| No |  | 10 / 63 (16%) |
| Yes |  | 53 / 63 (84%) |
| **Ann Arbor's classification, n / N (%)** | 124 |  |
| 1 |  | 1 / 124 (0.8%) |
| 2 |  | 2 / 124 (1.6%) |
| 3 |  | 5 / 124 (4.0%) |
| 4 |  | 116 / 124 (94%) |
| **Medullar infiltration, n / N (%)** | 123 | 84 / 123 (68%) |
| **CNS involvement, n / N (%)** | 118 | 61 / 118 (52%) |
| **Reason of Admission, n / N (%)** | 119 |  |
| Acute Kidney Injury |  | 14 / 119 (12%) |
| Acute Respiratory Failure |  | 13 / 119 (11%) |
| Coma |  | 1 / 119 (0.8%) |
| Monitoring |  | 50 / 119 (42%) |
| Other cause |  | 11 / 119 (9.2%) |
| Shock |  | 7 / 119 (5.9%) |
| Tumor Lysis Syndrome |  | 23 / 119 (19%) |
| **SOFA Score - Admission, Median [Q1, Q3]** | 124 | 2.00 [1.00, 5.00] |
| **Chemotherapy in ICU, n / N (%)** | 124 | 104 / 124 (84%) |
| **Tumor lysis syndrome, n / N (%)** | 124 | 73 / 124 (59%) |
| **Hemophagocytic Syndrome, n / N (%)** | 124 | 4 / 124 (3.2%) |
| **Aplasia, n / N (%)** | 124 | 41 / 124 (33%) |
| **Vasopressors, n / N (%)** | 124 | 28 / 124 (23%) |
| **Mechanical ventilation, n / N (%)** | 124 | 32 / 124 (26%) |
| **Non-invasive ventilation, n / N (%)** | 124 | 6 / 124 (4.8%) |
| **RRT, n / N (%)** | 124 | 65 / 124 (52%) |
| **Length of ICU stay (days), Median [Q1, Q3]** | 124 | 5.00 [2.50, 9.00] |
| **Delay diagnosis - ICU admission (days), Median [Q1, Q3]** | 124 | 4.00 [0.00, 13.00] |
| **Year of Admission, n / N (%)** | 124 |  |
| 2014 and before |  | 79 / 124 (64%) |
| 2015 and after |  | 45 / 124 (36%) |

## **Table S2:** Progression free survival probability at 3, 6 and 12 months by subgroup

| **Characteristic**^1^ | **3 Months**^1^ | **6 Months**^1^ | **12 Months**^1^ | **p-value**^1^ |
| --- | --- | --- | --- | --- |
| **HIV infection** |  |  |  | 0.002 |
| HIV - | 76% (65%, 89%) | 70% (58%, 84%) | 68% (56%, 82%) |  |
| HIV + | 58% (48%, 71%) | 46% (36%, 59%) | 39% (29%, 52%) |  |
| **Sex** |  |  |  | 0.82 |
| Male | 63% (54%, 73%) | 56% (47%, 66%) | 50% (41%, 61%) |  |
| Female | 76% (61%, 95%) | 55% (39%, 79%) | 51% (35%, 75%) |  |
| **Diagnosis** |  |  |  | 0.67 |
| Burkitt's lymphoma | 60% (51%, 72%) | 54% (44%, 66%) | 50% (40%, 62%) |  |
| LAL-3 | 74% (62%, 88%) | 59% (46%, 75%) | 52% (39%, 69%) |  |
| **Vasopressors** |  |  |  | <0.001 |
| Vasopressors - | 79% (72%, 88%) | 67% (58%, 77%) | 60% (51%, 71%) |  |
| Vasopressors + | 18% (8.1%, 40%) | 18% (8.1%, 40%) | 18% (8.1%, 40%) |  |
| **Mechanical Ventilation** |  |  |  | <0.001 |
| MV - | 79% (72%, 88%) | 66% (57%, 77%) | 62% (52%, 72%) |  |
| MV + | 25% (14%, 46%) | 25% (14%, 46%) | 18% (8.3%, 38%) |  |
| **RRT** |  |  |  | <0.001 |
| RRT - | 83% (74%, 93%) | 71% (60%, 84%) | 67% (56%, 81%) |  |
| RRT + | 49% (39%, 63%) | 42% (31%, 56%) | 35% (25%, 49%) |  |
| **Tumor Lysis Syndrome** |  |  |  | <0.001 |
| TLS - | 80% (70%, 92%) | 74% (63%, 87%) | 72% (61%, 86%) |  |
| TLS + | 55% (45%, 68%) | 42% (32%, 55%) | 35% (26%, 48%) |  |
| **Tumor Lysis Syndrome & RRT** |  |  |  | 0.001 |
| TLS- | 80% (70%, 92%) | 74% (63%, 87%) | 72% (61%, 86%) |  |
| TLS+/RRT- | 69% (48%, 99%) | 36% (17%, 77%) | 27% (10%, 69%) |  |
| TLS+/RRT+ | 52% (41%, 66%) | 43% (33%, 58%) | 37% (26%, 51%) |  |
| ^1^Survival rates reported as % (95% CI) | | | | |

## **Table S3**: Patients’ characteristics according to HIV status

| **Variable** | **N** | **No HIV infection** N = 52 | **HIV infection**  N = 75 | **p-value**^1^ |
| --- | --- | --- | --- | --- |
| **Age, Median (Q1, Q3)** | 127 | 41.00 (27.00, 60.50) | 48.00 (38.00, 56.00) | 0.20 |
| **Male Sex, n / N (%)** | 127 | 37 / 52 (71%) | 65 / 75 (87%) | 0.031 |
| **Comorbidities, n / N (%)** | 127 |  |  | 0.76 |
| Chronic Kidney Disease |  | 1 / 52 (1.9%) | 1 / 75 (1.3%) |  |
| Cirrhosis |  | 1 / 52 (1.9%) | 0 / 75 (0%) |  |
| COPD |  | 1 / 52 (1.9%) | 1 / 75 (1.3%) |  |
| No |  | 39 / 52 (75%) | 54 / 75 (72%) |  |
| Other |  | 10 / 52 (19%) | 19 / 75 (25%) |  |
| **Diagnosis, n / N (%)** | 127 |  |  | 0.42 |
| Burkitt |  | 31 / 52 (60%) | 50 / 75 (67%) |  |
| L3-ALL |  | 21 / 52 (40%) | 25 / 75 (33%) |  |
| **Type of biopsy, n / N (%)** | 127 |  |  | 0.90 |
| Lymph Node |  | 10 / 52 (19%) | 16 / 75 (21%) |  |
| Medullar |  | 20 / 52 (38%) | 26 / 75 (35%) |  |
| Other |  | 22 / 52 (42%) | 33 / 75 (44%) |  |
| **Caryotype, n / N (%)** | 79 |  |  | 0.28 |
| No medullar involvement |  | 2 / 32 (6.3%) | 10 / 47 (21%) |  |
| No translocation |  | 5 / 32 (16%) | 9 / 47 (19%) |  |
| t(8;14) |  | 23 / 32 (72%) | 26 / 47 (55%) |  |
| t(8;22) |  | 2 / 32 (6.3%) | 2 / 47 (4.3%) |  |
| **cMyc rearrangement - FISH, n / N (%)** | 63 |  |  | 0.73 |
| No |  | 3 / 24 (13%) | 7 / 39 (18%) |  |
| Yes |  | 21 / 24 (88%) | 32 / 39 (82%) |  |
| **Ann Arbor's classification, n / N (%)** | 127 |  |  | 0.76 |
| 1 |  | 0 / 52 (0%) | 1 / 75 (1.3%) |  |
| 2 |  | 1 / 52 (1.9%) | 1 / 75 (1.3%) |  |
| 3 |  | 3 / 52 (5.8%) | 2 / 75 (2.7%) |  |
| 4 |  | 48 / 52 (92%) | 71 / 75 (95%) |  |
| **Medullar infiltration, n / N (%)** | 124 | 34 / 51 (67%) | 51 / 73 (70%) | 0.71 |
| **CNS involvement, n / N (%)** | 120 | 18 / 50 (36%) | 44 / 70 (63%) | 0.004 |
| **Reason of Admission, n / N (%)** | 121 |  |  | 0.71 |
| Acute Kidney Injury |  | 6 / 48 (13%) | 8 / 73 (11%) |  |
| Acute Respiratory Failure |  | 5 / 48 (10%) | 8 / 73 (11%) |  |
| Coma |  | 0 / 48 (0%) | 1 / 73 (1.4%) |  |
| Monitoring |  | 24 / 48 (50%) | 28 / 73 (38%) |  |
| Other cause |  | 2 / 48 (4.2%) | 9 / 73 (12%) |  |
| Shock |  | 3 / 48 (6.3%) | 4 / 73 (5.5%) |  |
| Tumor Lysis Syndrome |  | 8 / 48 (17%) | 15 / 73 (21%) |  |
| **SOFA Score - Admission, Median (Q1, Q3)** | 127 | 2.00 (1.00, 4.00) | 3.00 (1.00, 5.00) | 0.21 |
| **Chemotherapy in ICU, n / N (%)** | 127 | 47 / 52 (90%) | 60 / 75 (80%) | 0.11 |
| **Tumor lysis syndrome, n / N (%)** | 127 | 33 / 52 (63%) | 42 / 75 (56%) | 0.40 |
| **Hemophagocytic Syndrome, n / N (%)** | 127 | 0 / 52 (0%) | 4 / 75 (5.3%) | 0.14 |
| **Aplasia, n / N (%)** | 127 | 14 / 52 (27%) | 28 / 75 (37%) | 0.22 |
| **Vasopressors, n / N (%)** | 127 | 9 / 52 (17%) | 19 / 75 (25%) | 0.28 |
| **Mechanical ventilation, n / N (%)** | 127 | 9 / 52 (17%) | 23 / 75 (31%) | 0.088 |
| **Non-invasive ventilation, n / N (%)** | 127 | 4 / 52 (7.7%) | 2 / 75 (2.7%) | 0.23 |
| **RRT, n / N (%)** | 127 | 28 / 52 (54%) | 39 / 75 (52%) | 0.84 |
| **Length of ICU stay (days), Median (Q1, Q3)** | 127 | 4.00 (2.00, 10.00) | 5.00 (3.00, 9.00) | 0.52 |
| **Delay diagnosis - ICU admission (days), Median (Q1, Q3)** | 127 | 5.00 (0.00, 12.50) | 3.00 (0.00, 13.00) | 0.46 |
| **Status at 90 days, n / N (%)** | 124 |  |  | 0.056 |
| Alive |  | 38 / 50 (76%) | 44 / 74 (59%) |  |
| Deceased |  | 12 / 50 (24%) | 30 / 74 (41%) |  |
| **Year of Admission, n / N (%)** | 127 |  |  | 0.87 |
| 2014 and before |  | 34 / 52 (65%) | 48 / 75 (64%) |  |
| 2015 and after |  | 18 / 52 (35%) | 27 / 75 (36%) |  |
| ^1^Wilcoxon rank sum test; Pearson's Chi-squared test; Fisher's exact test | | | | |

## **Table S4:** Risk factors for one year mortality using a multivariate Cox model with an interaction term between RRT and TLS

| **Characteristic** | **HR (95% CI)** | **p-value** |
| --- | --- | --- |
| **HIV infection^1^** | 2.39 (1.31 to 4.36) | **0.005** |
| **Male Sex^1^** | 1.84 (0.88 to 3.84) | 0.10 |
| **Age (per 5 years)^1^** | 1.14 (1.03 to 1.27) | **0.010** |
| **Vasopressors^1^** | 4.02 (1.78 to 9.09) | **<0.001** |
| **Mechanical Ventilation^1^** | 2.07 (0.95 to 4.49) | 0.066 |
| **RRT^1^** | 1.93 (0.56 to 6.68) | 0.30 |
| **Tumor Lysis Syndrome^1^** | 7.39 (2.82 to 19.3) | **<0.001** |
| **Year of Admission^1^** |  |  |
| 2014 and before^1^ | — |  |
| 2015 and after^1^ | 0.48 (0.26 to 0.91) | **0.024** |
| **RRT * Tumor Lysis Syndrome^1^** | 0.26 (0.06 to 1.11) | 0.069 |
| ^1^Exploratory interaction model — complete case analysis (n = 127, events = 61). | | |
| Abbreviations: CI = Confidence Interval, HR = Hazard Ratio | | |

## **Table S5:** Proportional hazards assumption — Schoenfeld residuals test

| **Variable** | **Chi-square** | **df** | **p-value** | **p_flag** |
| --- | --- | --- | --- | --- |
| HIV infection | 0.836 | 1 | 0.361 |  |
| Male Sex | 0.159 | 1 | 0.690 |  |
| Age (per 5 years) | 1.384 | 1 | 0.240 |  |
| Vasopressors | 7.419 | 1 | **0.006** | ** |
| Mechanical Ventilation | 3.639 | 1 | 0.056 |  |
| Tumor Lysis Syndrome | 12.113 | 1 | **0.001** | ** |
| Year of Admission | 1.361 | 1 | 0.243 |  |
| ***Global*** | ***22.151*** | ***7*** | ***0.002*** | ******** |

## Table S6: Sensitivity analysis — Cox model restricted to baseline predictors only

| **Variable** | **HR (95% CI)** | **p-value** |
| --- | --- | --- |
| **HIV infection^1^** | 2.09 (1.18 to 3.71) | **0.012** |
| **Age (per 5 years)^1^** | 1.16 (1.05 to 1.28) | **0.002** |
| **SOFA score at admission (per point)^1^** | 1.12 (1.06 to 1.18) | **<0.001** |
| **Year of Admission^1^** |  |  |
| 2014 and before^1^ | — |  |
| 2015 and after^1^ | 0.52 (0.29 to 0.91) | **0.023** |
| ^1^Sensitivity analysis restricted to baseline variables only (n = 127, events = 61). | | |
| Abbreviations: CI = Confidence Interval, HR = Hazard Ratio | | |

## **Table S7**: Variance Inflation Factors (VIF) analysis

| **Variable** | **VIF** | **Interpretation** |
| --- | --- | --- |
| RRT | 2.137 | Moderate |
| Vasopressors | 2.007 | Moderate |
| Mechanical Ventilation | 1.959 | No |
| Tumor Lysis Syndrome | 1.948 | No |
| Male Sex | 1.301 | No |
| Year of Admission | 1.178 | No |
| Age (per 5 years) | 1.104 | No |
| HIV infection | 1.090 | No |
| VIF < 5 is considered as low colinearity | | |
